# Impact of pyriproxyfen on virus behavior: implications for pesticide-induced virulence and mechanism of transmission

**DOI:** 10.1186/s12985-020-01378-y

**Published:** 2020-07-06

**Authors:** Paula A. Faria Waziry, Aarti Raja, Chloe Salmon, Nathalia Aldana, Sruthi Damodar, Andre Rinaldi Fukushima, Bindu S. Mayi

**Affiliations:** 1 Dr. Kiran C. Patel College of Osteopathic Medicine, Nova Southeastern University, 3400 Gulf to Bay Blvd, Clearwater, FL 33759 USA; 2 Department of Biological Sciences, Halmos College of Arts and Sciences, Nova Southeastern University, Fort Lauderdale, FL 33314 USA; 3 Plymouth University, 3 Endsleigh Place, Drake Circus, Plymouth, England PL4 8AA; 4 Dr. Kiran C. Patel College of Osteopathic Medicine, Nova Southeastern University, 3200 S. University Dr, Fort Lauderdale, FL 33328 USA; 5 Department of Pathology, School of Veterinary Medicine and Animal Science, University of Sao Paulo, São Paulo, Brazil

**Keywords:** Neurovirulence, Pyriproxyfen, Pesticides, Flavivirus replication, Extracellular vesicles, Wolbachia, Mosquito control

## Abstract

**Background:**

More than 3 years since the last Zika virus (ZIKV) outbreak in Brazil, researchers are still deciphering the molecular mechanisms of neurovirulence and vertical transmission, as well as the best way to control spread of ZIKV, a flavivirus. The use of pesticides was the main strategy of mosquito control during the last ZIKV outbreak.

**Methods:**

We used vesicular stomatitis virus (VSV) tagged with green fluorescent protein (GFP) as our prototypical virus to study the impact of insecticide pyriproxyfen (PPF). VZV-GFP infected and uninfected Jurkat, HeLa and trophoblast cells were treated with PPF and compared to untreated cells (control). Cell viability was determined by the MTT assay. Cell morphology, presence of extracellular vesicles (EVs), virus infection/GFP expression as well as active mitochondrial levels/localization were examined by confocal microscopy.

**Results:**

PPF, which was used to control mosquito populations in Brazil prior to the ZIKV outbreak, enhances VSV replication and has cell membrane-altering properties in the presence of virus. PPF causes enhanced viral replication and formation of large EVs, loaded with virus as well as mitochondria. Treatment of trophoblasts or HeLa cells with increasing concentrations of PPF does not alter cell viability, however, it proportionately increases Jurkat cell viability. Increasing concentrations of PPF followed by VSV infection does not interfere with HeLa cell viability. Both Jurkats and trophoblasts show proportionately increased cell death with increased concentrations of PPF in the presence of virus.

**Conclusions:**

We hypothesize that PPF disrupts the lipid microenvironment of mammalian cells, thereby interfering with pathways of viral replication. PPF lowers viability of trophoblasts and Jurkats in the presence of VSV, implying that the combination renders immune system impairment in infected individuals as well as enhanced vulnerability of fetuses towards viral vertical transmission. We hypothesize that similar viruses such as ZIKV may be vertically transmitted via EV-to-cell contact when exposed to PPF, thereby bypassing immune detection. The impact of pesticides on viral replication must be fully investigated before large scale use in future outbreaks of mosquito borne viruses.

## Background

Mosquito borne viral diseases such as Dengue fever, West Nile virus encephalitis, Chikungunya, Yellow fever, and Zika, are diseases that present treatment challenges. They also create a major public health crisis when they are detected in epidemic proportions. In the absence of vaccines and antiviral drugs for most of these viruses, a major focus remains on mosquito or vector eradication to limit disease/virus transmission. The elimination of pathogen transmitting mosquito species *Aedes aegypti* and *Aedes albopictus* has many challenges. *Aedes* finds a natural habitat in crowded tropical cities and can easily propagate indoors and/or outdoors in small bodies of water [1]. The initial approach in the United States in the 2015/2016 Zika virus (ZIKV) outbreak was to spray insecticides and larvicides in all areas suspected of local transmission [2]. That strategy appears to have reduced mosquito numbers, thereby halting the spread of ZIKV. The outlook was worse for hard-hit Brazil, where the inefficient and largely ineffective implementation of a comprehensive *Aedes aegypti* control plan did not prevent the massive outbreak of ZIKV that was reported [3].

It has been suggested that in Brazil, one of the agents used for mosquito control was the pesticide, pyriproxyfen (2-[1-methyl-2-(4-phenoxyphenoxy) ethoxy] pyridine or PPF for short) [4]. Questions remain about the impact of using PPF as a means of mosquito control. To date, no documented studies have explored the possibility that PPF could interfere with mammalian cell membrane structure and function, thereby interfering with viral replication as well as mode of transmission. The life cycle of enveloped cytoplasmic viruses, which include orthomyxovirus (e.g. VSV) and flaviviruses (e.g. ZIKV), is dependent on cellular lipid metabolism, which in turn influences the steps of viral attachment, fusion and budding [5]. Host lipid-modifying pathways have been shown to be utilized by distantly related positive-sense RNA viruses for formation of replication sites [6]. Budding of newly replicated VSV is dependent on the integrity of membrane microdomains [7]. Although PPF is generally recognized as safe, we hypothesize that PPF could disrupt the lipid microenvironment of mammalian cells, thereby interfering with pathways of viral replication when those cells are infected. It is worth noting that the brain, which can be targeted by viruses, is largely composed of lipids [8–10]. The chemical structure of PPF mimics insect juvenile hormone (JH), is lipid-soluble, and causes serious disruptions in hormone pathways, affecting development and reproduction of insects, insect behavior, pheromone production as well as adult morphogenesis [11]. The lipophilic nature of PPF hints at a potential influence on membrane lipid composition of cells that are exposed to it, thereby influencing membrane processes such as formation of extracellular vesicles (EV), viral entry, replication and budding.

We have tested the effects of PPF on viability of: (a) primary human placental trophoblasts, which play a critical role as a physical barrier against teratogenic virus transmission [12, 13]; (b) HeLa cell line, derived from uterine epithelial tissue, which is affected by viruses and can utilize non-conventional cell-to-cell transmission [14] and (c) Jurkat cell line, derived from human lymphocytes (T-cells), which was chosen due to its importance as immune system mediator and due to previously shown resistance to toxic/insecticidal compounds [15]. Cells were infected with a prototypical virus, the Vesicular Stomatitis Virus (VSV) at MOI of 1.0 for viability assays and at MOI of 0.001 for immunostaining. VSV is considered teratogenic and neurotropic [16, 17]. Although VSV infections are mainly asymptomatic in humans, it can cause flu-like symptoms and has been shown to be capable of infecting brain cells and causing encephalitis [18].

Our results show a preferential impact of PPF on virus infected cells. In the absence of viral infection, treatment of trophoblasts or HeLa cells with increasing concentrations of PPF does not alter cell viability. Treatment of Jurkat cells with PPF, however, proportionately increased Jurkat cell viability. Similar results using an antimicrobial/insecticidal agent had previously been shown with Jurkat cells and HeLa cells exposed to scorpion venom [15]. Increasing concentrations of PPF followed by VSV infection does not interfere with HeLa cell viability. In the presence of virus, both Jurkat cells and trophoblasts show proportionately increased cell death with increased concentrations of PPF. VSV-GFP infected Jurkat cells and HeLa cells treated with PPF showed the presence of EV that were freestanding as well as in contact with cells. These types of structures could potentially result in quick transmission of virus among cells within the host as well as vertical transmission. We show for the first time that pesticide treatment decreases viability of infected cells (Jurkat cells and trophoblasts) and promotes increased viral replication in newly detected EVs. A potential consequence of using a pesticide such as PPF is its impact on mammalian cell structure, with a downstream enhancement of replication and transmission of a virus that is heavily dependent on its host cell membrane integrity. Further studies using viruses such as ZIKV are warranted to better understand the impact of pesticide use on viral replication.

## Methods

### Cell culture, pesticide treatment and VSV infection

The following human cells were obtained from the American Tissue Culture Collection: Jurkat (T-cell derived cell line) (ATCC, clone E6–1), HeLa (clone CCL-2), and trophoblasts (ATCC, CRL-3271). Trophoblasts and Jurkat cells were grown in RPMI media (Cellgro, Fisher Scientific), while HeLa cells were grown in DMEM media (Cellgro, Fisher Scientific). Both types of media contained 10% HyClone™ Fetal Bovine Serum (FBS, Fisher Scientific), supplemented with 1% HyClone™ antibiotic/antimycotic solution (Fisher Scientific) in an atmosphere of 5% CO_2_, at 37 °C. For immunostaining experiments, cells were treated for 12 h with 0.01 μg/mL of pyriproxyfen (PPF; Sigma-Aldrich), a concentration equivalent to that used in Brazil to inhibit mosquito propagation in drinking water. The Control cells were treated for 12 h with equivalent concentration of PPF solvent dimethylsulfoxide (DMSO). Infections with VSV-GFP virus (vesicular stomatitis virus, Indiana strain, fused with green fluorescent protein, a gift from Dr. Glen Barber, University of Miami Sylvester Cancer Center) were performed as previously described [19]. Briefly, cells were plated on poly-L-lysine treated glass cover slips placed on 6-well plates and infected for 16 h with VSV tagged with green fluorescent protein (VSV-GFP) without FBS or antimicrobials, at a very low multiplicity of infection (MOI) of 0.001.

### Cell viability (MTT) assay

Cells were seeded at a density of 1 × 10^4^ in 96-well plates and allowed to settle overnight. Cells were then either not treated (control) or treated with several concentrations of PPF (from 0.001 to 10.0 μg/mL) and incubated overnight. Next day, cells were infected with VSV-GFP at MOI of 1.0 and again incubated overnight. Subsequently, cells were treated with MTT (3-(4,5-Dimethylthiazol-2-yl)-2,5-diphenyltetrazolium bromide (Sigma-Aldrich, MO) dissolved in PBS (stock solution is 5 mg/ml) at a final concentration of 5 μg/ml. After incubation for approximately 2 h at 37 °C, 5% CO2, the MTT-treated media was aspirated from adherent cells (trophoblasts and HeLa cells), which were subsequently exposed to 200 μl Dimethyl sulfoxide (DMSO) (Sigma-Aldrich, MO) per well. Cell density was measured with a microplate-reader at a wavelength of 570 nm. Suspension cells (Jurkat cells) were centrifuged at low speed (180xG) and the media was carefully aspirated in order not to disturb sedimented cells. Addition of 200 μl DMSO and a cell density reading at 570 nm followed as above. We compared the viability of cells treated with PPF and subsequently either uninfected or infected with VSV-GFP. All experiments were carried out in multiples of 8. Cell viability percentages were estimated by dividing the absorbance values of treated cells with that of control cells.

### Immunostaining and confocal microscopy

Cells were treated as above with PPF, infected (or not) with VSV-GFP at a MOI of 0.001and fixed with 4% formaldehyde (Sigma-Aldrich) for 8 min at room temperature. Cells were then washed thrice in PBS and stained with Hoescht 33,258 (Invitrogen) for 5 min in a humidifying chamber at room temperature. Cells were washed thrice with PBS and mounted on glass slides using ProLong Antifade Gold reagent (Invitrogen). HeLa cells were obtained from ATCC (clone CCL-2), grown in DMEM media containing 10% HyClone™ FBS supplemented with 1% HyClone™ antibiotic/antimycotic solution, in an atmosphere of 5% CO_2_ at 37 °C. HeLa cells were treated with PPF, DMSO and VSV-GFP as described above for Jurkat cells. For mitochondria-labeling experiments, cells were treated with PPF and VSV-GFP as described above. These cells were subsequently treated with MitoTracker Orange for 30 min, then fixed for 10 min with cold methanol at − 20 °C according to manufacturer’s instructions (Invitrogen). Samples were examined on a Zeiss LSM880 confocal microscope using Zen software. The above experiments were performed three independent times and in triplicate, each time. Fluorescence profile analysis of VSV-GFP, mitochondria, nuclear staining and cell morphology were performed using Zen software (Zeiss).

## Results

### PPF appears to decrease viability of VSV-GFP infected trophoblasts and Jurkat cells (Fig. 1)

We conducted viability tests of trophoblasts, HeLa cells and Jurkat cells treated with varying concentrations of PPF from 0.001 to 10.0 μg/mL These cells were subsequently uninfected or infected with VSV (a prototypical enveloped virus that replicates in the cytoplasm of cells, similarly to ZIKV) fused to Green fluorescence protein (VSV-GFP) at a MOI of 1.0. PPF alone does not appear to greatly affect viability of trophoblasts, HeLa cells, or Jurkat cells. Infection with VSV-GFP reduces cell viability of all three cell lines. Increasing concentrations of PPF affects viability of infected trophoblasts. The most dramatic results are seen with HeLa cells, where cell viability drastically decreases in infected cells. Increasing the concentration of PPF does not further decrease viability of infected HeLa cells. In the case of infected Jurkat cells, increasing concentrations of PPF decreases the viability of infected cells.

**Fig. 1:**
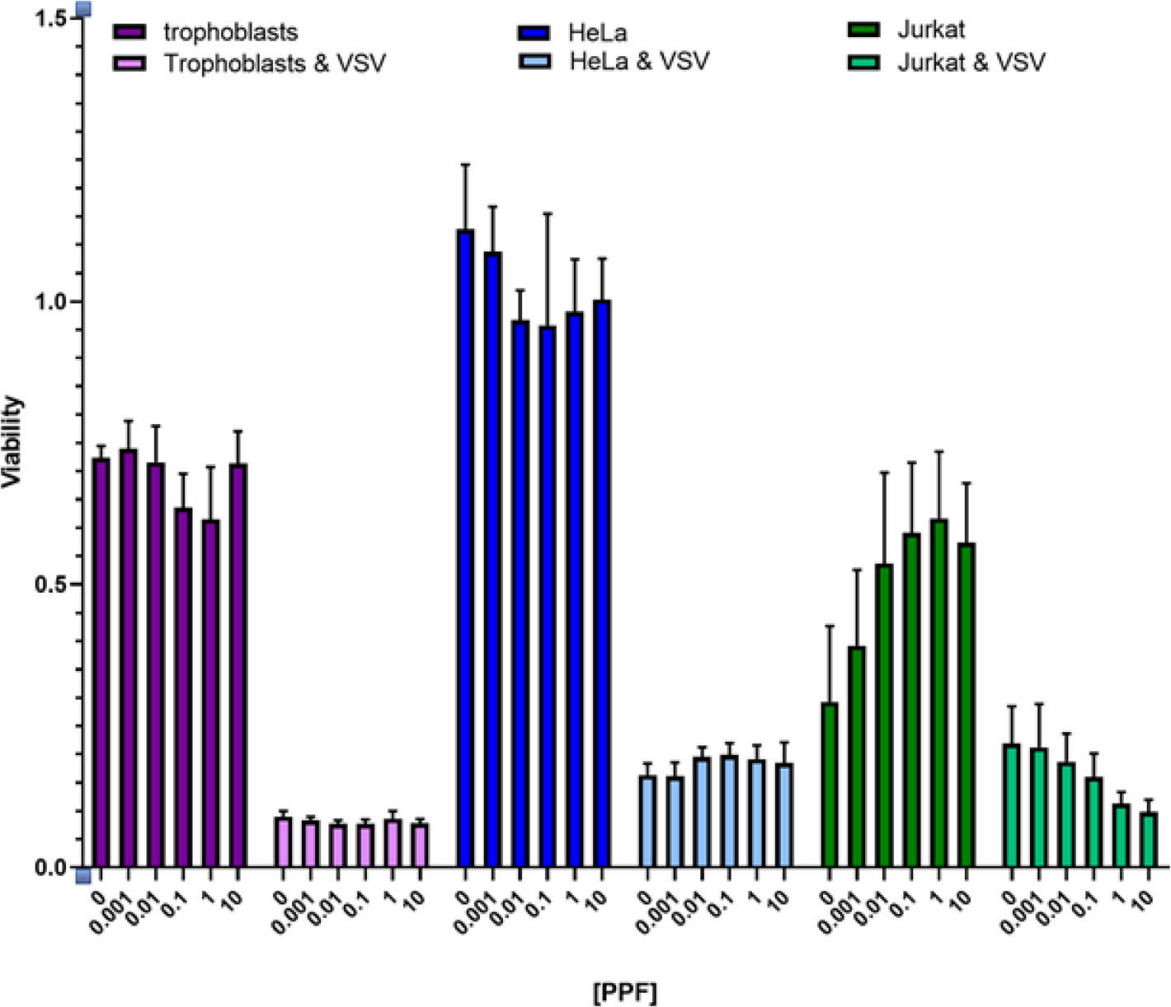
Cell viability (MTT) assay. **a** Trophoblasts treated with increasing concentrations of PPF (μg/mL). **b** Trophoblasts infected with VSV-GFP (MOI of 1.0) and treated with increasing concentrations of PPF (μg/mL). **c** HeLa cells treated with increasing concentrations of PPF (μg/mL). **d** HeLa cells infected with VSV-GFP (MOI of 1.0) and treated with increasing concentrations of PPF (μg/mL). **e** Jurkat cells treated with increasing concentrations of PPF (μg/mL). **f** Jurkat cells infected with VSV-GFP (MOI of 1.0) and treated with increasing concentrations of PPF (μg/mL)

### PPF treated cells infected with VSV-GFP show formation of large extracellular vesicle (EV) filled with virus (Fig. 2)

**Fig. 2:**
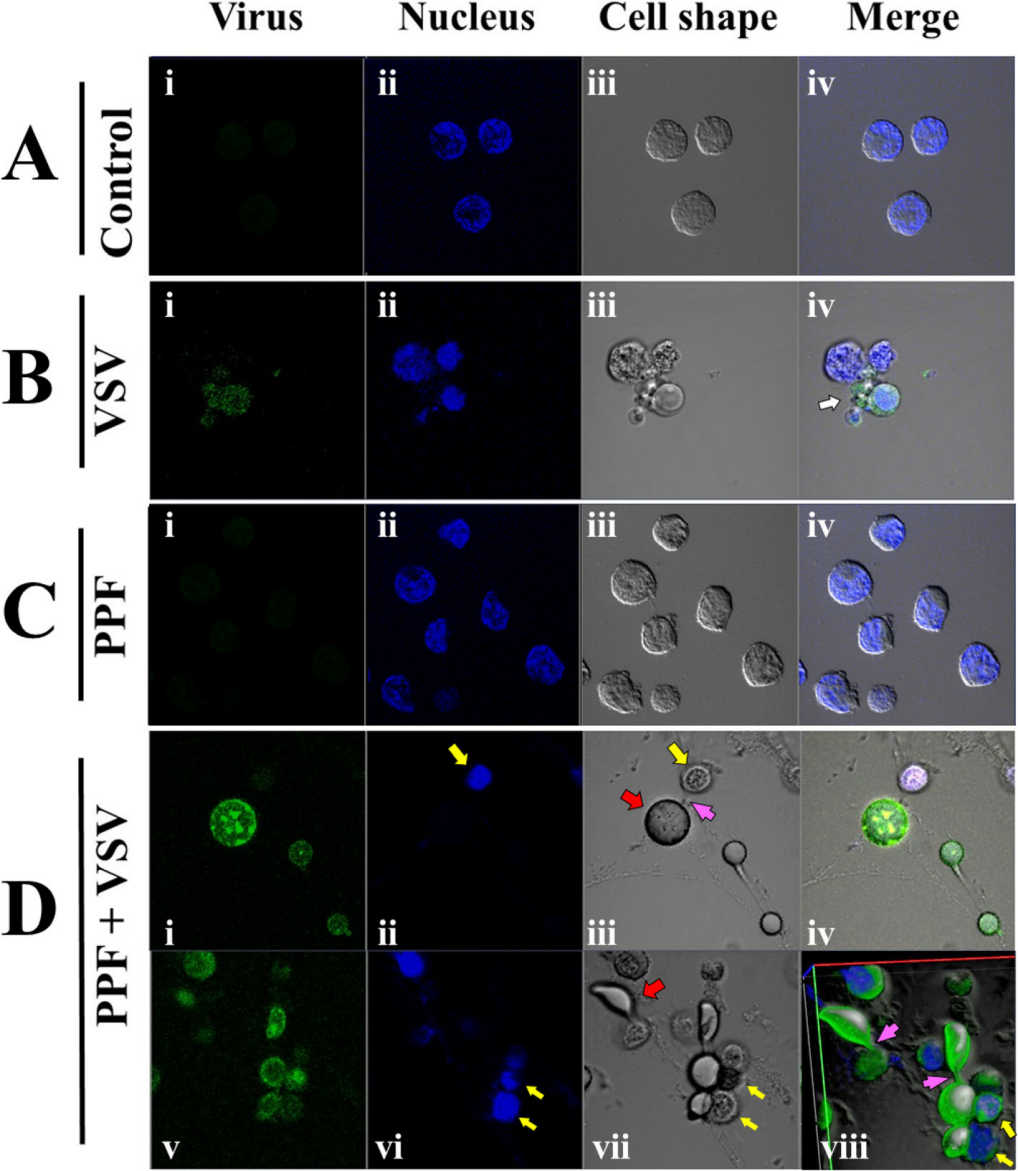
**a** Jurkat cells untreated with PPF and uninfected with VSV-GFP. **b** Jurkat cells infected with VSV-GFP. **c** Jurkat cells treated with PPF. **d** Jurkat cells treated with PPF and infected with VSV-GFP. In all cases, panel i is for green fluorescence corresponding to VSV-GFP replication; panel ii shows Hoecsht 33,258 stained nuclei of Jurkat cells; panel iii shows DIC microscopy of cells; panel iv reveals a merged image of panels i, ii and iii

Based on our hypothesis that PPF disrupts the membrane microenvironment of mammalian cells, thereby interfering with pathways of viral replication, we exposed Jurkat cells to PPF in vitro, at concentrations similar to those used for treatment of drinking water in Brazil. Control Jurkat cells, untreated with PPF and uninfected with VSV-GFP are shown in Fig. 2a. As expected, there is a lack of green fluorescence, a marker for the presence and replication level of virus (Fig. 2ai). Hoecsht staining reveals presence of nuclei, distinguishing cells (blue structures, Fig. 2aii) from vesicles, while differential interference contrast (DIC) microscopy (Fig. 2aiii) reveals normal morphology of cells. A merged image of i, ii and iii (Fig. 2aiv) shows Jurkat cells with normal morphology, stained nuclei and no virus.

**Fig. 3:**
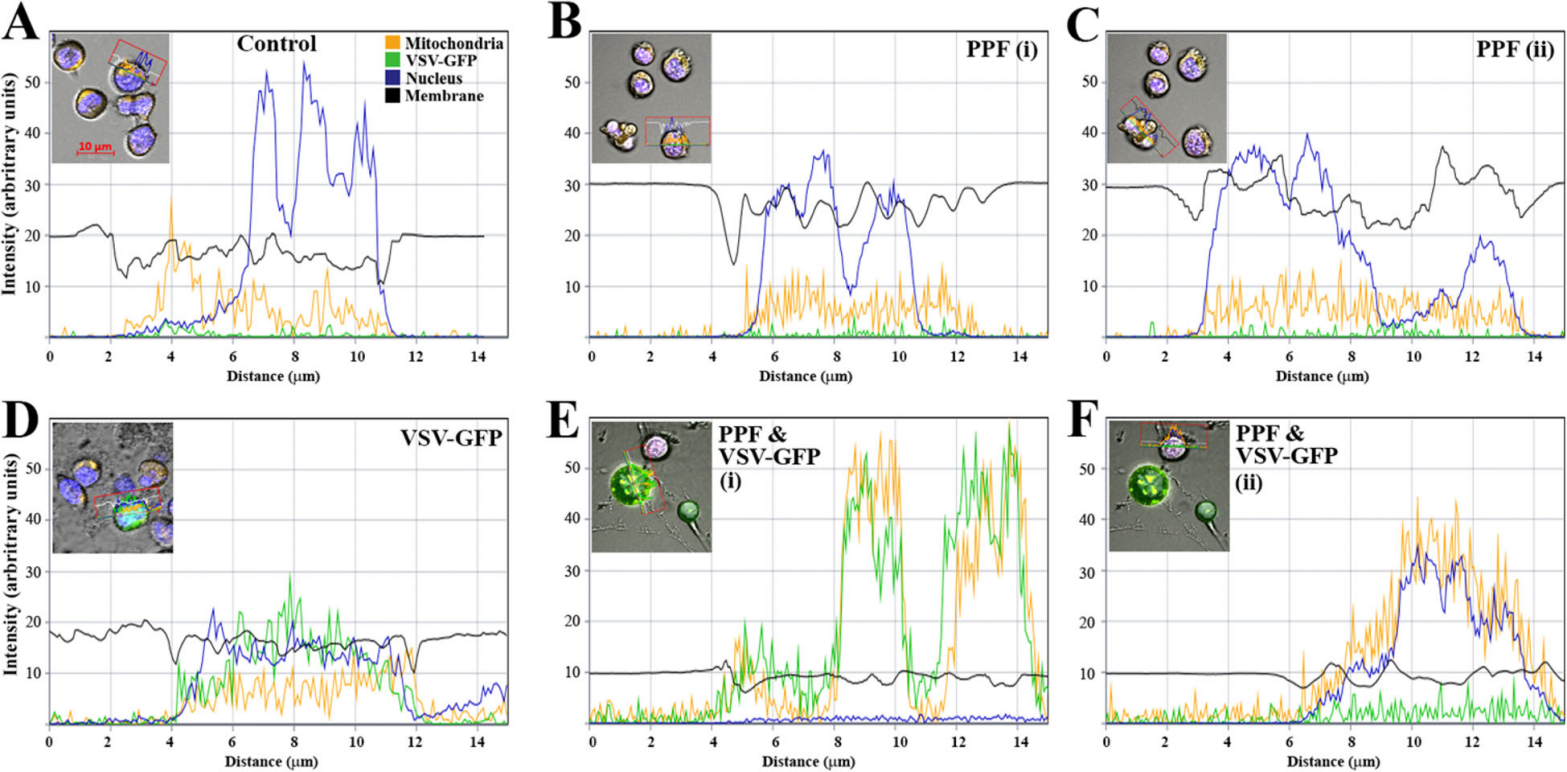
Fluorescence profile of VSV-GFP (green channel), nuclei (blue channel), mitochondria (orange channel) and cell membrane morphology (black graph). Jurkat cells were analyzed by quantitative confocal microscopy. **a** Control Jurkat cells untreated with PPF and uninfected with VSV-GFP. **b** cells treated with PPF alone, with fluorescence profile of an intact cell (shown within the red box in the inset). **c** cells treated with PPF alone with fluorescence profile of an apoptotic cell (shown within the red box in the inset). **d** cells infected with VSV-GFP at a multiplicity of infection of 0.001 with fluorescence profile of a cell (shown within the red box in the inset). **e** and **f** cells were first treated with PPF, then infected with VSV-GFP. **e** shows the fluorescence profile of a large EV (shown within the red box in the inset), while **f** shows the fluorescence profile of a nearby infected cell

Figure 2b represents cells untreated with PPF and infected with VSV-GFP. As expected, we see the presence of virus within cells, denoted by green fluorescence representing VSV-GFP (Fig. 2bi). Host cells supporting VSV-GFP replication eventually undergo apoptosis, as revealed by Fig. 2bii, which shows stained nuclei with a fragmented cell morphology that indicates initiation of apoptosis. DIC microscopy (Fig. 2biii) further confirms the apoptotic cell morphology. A merging of images i, ii and iii reveals the presence of VSV-GFP within nucleated cells (Fig. 2biv), again as expected. A few EVs were also observed, a phenotype that is normal for Jurkat cells (shown by white arrow in Fig. 2div) [20]. Some of the EVs showed the presence of virus, albeit very little, as reflected by the low intensity of green fluorescence measured by quantitative confocal microscopy (Fig. 3).

Our next step was to analyze Jurkat cells treated with PPF and uninfected with virus. As expected, there is a lack of green fluorescence (Fig. 2ci) and most cells appear to have normal morphology (Fig. 2cii, iii, iv). Analysis of PPF-treated and VSV-GFP infected cells revealed the presence of EVs packed with virus, as revealed by green fluorescence (Fig. 2di and iv), with the intensity of green fluorescence being proportional to the relative amount of viruses. The concentrated intensity of green fluorescence at the periphery of a large EVs shows high expression and localization of VSV-GFP at the membrane of the EV (Fig. 2di and v). The EV is a structure distinct from the Jurkat cell which is revealed by the presence of blue stained nucleus (yellow arrow, Fig. 2dii) adjacent to the EV. DIC microscopy (Fig. 2diii) shows the normal morphology of Jurkat cells (yellow arrow) and also depicts the shape of the large round EV devoid of a nucleus (red arrow). Jurkat cells appear smaller than the VSV-filled EV (Fig. 2diii, red arrow) and the two structures seem interconnected by a tubule (pink arrow). VSV-GFP packed EV vary in size and shape. Some of these structures appear attached to cells (pink arrows, Figs. 2diii and dviii) while others are free standing. Figure 2dv-viii represent a different field of view and show what appear to be vesicles of various sizes and shapes, seemingly emerging from cells (red arrow, Fig. 2dvii), connected by tubules (pink arrows). Figure 2dviii shows more intense green fluorescence (corresponding to more viruses) in attached EVs than in surrounding Jurkat cells (yellow arrow).

The variety and number of EVs suggests their presence to be an active process needing the use of mitochondria, the energy powerhouse of the cell. Our next step was to investigate the presence of active mitochondria in these structures, knowing that VSV interacts/co-localizes with mitochondria [21, 22].

### PPF treated cells infected with VSV-GFP accumulate viruses as well as mitochondria in extracellular vesicles (EVs) (Fig. 3)

VSV is known to interfere with mitochondria, diverting cellular energy metabolism towards enhancing viral replication and ultimately inducing cell apoptosis [22]. We subjected Jurkat cells treated or untreated with PPF with or without VSV-GFP infection, to quantitative confocal microscopy. We assessed the fluorescence intensity of active mitochondria (orange channel corresponding to Mito-tracker Orange), VSV-GFP (green channel) and nuclei (blue channel), with cell membranes (black graph) representing differential interference contrast. Figure 3a shows background fluorescence levels of Jurkat cells untreated with PPF and uninfected with VSV-GFP. The inset shows a few cells, stained blue for nuclei and orange for mitochondria, with the profiled fluorescence pattern representing the cell highlighted within the red box. As expected, there is a lack of green fluorescence. Orange fluorescence represents an uneventful state for the mitochondria. The peak in blue fluorescence represents nuclei of healthy cells, while the black graph represents structural integrity of the cell membrane.

Figure 3b and c both show PPF treated cells uninfected with virus, with the fluorescence profile representing the cell highlighted by the red box. The fluorescence profile seen in 3B is different from that seen in 3A and is likely an effect of PPF on the individual Jurkat cells. Figure 3c shows disturbed cell membrane (black graph) suggestive of apoptotic cell morphology (see inset), while Fig. 3b profiles an intact cell. Fluorescence analysis of cells untreated with PPF and infected with VSV-GFP showed cells filled with virus. When a large, green cell representing a Jurkat cell with VSV-GFP was analyzed by quantitative confocal microscopy (Fig. 3d), we saw viruses (green channel) co-localizing with nuclei (blue channel) and also with mitochondria (orange channel). Mitochondria (Fig. 3d) showed normal fluorescence, similar to the orange fluorescence profiles in Fig. 3a, b and c, implying background levels of engagement during VSV-GFP replication in Jurkat cells untreated with PPF.

PPF treated cells infected with VSV-GFP showed presence of virus within cells and EVs (Fig. 3e and f). Fluorescence analysis of a large EV (see inset, Fig. 3e) that appears attached to a cell, reveals presence of viruses (green channel), mitochondria (orange channel), and cell membrane (black graph), but no nuclear staining (blue channel), since the EVs are devoid of nuclei. The distribution of VSV-GFP inside these large EVs is very unusual, showing pockets of virus accumulated inside the vesicles as well as at the periphery or vesicle surface (quantitative analysis of Fig. 2di). The accumulated virus pockets within the Jurkat cells are also rich in mitochondria, with which the virus is co-localizing. Figure 3f shows the fluorescence profile of the cell (shown within the insert) attached to the large EV that was analyzed in Fig. 3e. Fluorescence analysis of this cell, treated with PPF and infected with virus reveals co-localization of nuclei (blue channel) and mitochondria (orange channel), with green fluorescence above the background levels seen in cells uninfected with virus (Figs. 3a, b and c). Higher green fluorescence in EVs (Fig. 3e) suggests higher viral load compared to cells untreated with PPF (Fig. 3d).

## Discussion

Investigators have shown the presence of EVs in HIV infected cells, enabling direct EV-to-cell transmission of the virus [23–25]. HIV transmission is faster and more efficient via EVs which promote direct cell contact [25]. The ability of a teratogenic virus such as VSV or ZIKV to be similarly transmitted by EVs could be an advantage in its pathogenesis and ability to infect the fetus. Based on the lipid-soluble, hormone-like chemical structure of PPF, we hypothesize that PPF is capable of disrupting cellular membrane composition and lipid metabolism. Our observations with VSV infection of PPF treated cells point to the possibility that formation of EVs might enable virus to be transmitted quickly in the presence of PPF, via EV-to-cell contact, thereby evading immune detection. Such EV-to-cell transmission might also be part of the mechanisms that allow vertical transmission of this teratogenic virus. EVs were also shown to be a quick and efficient mode of transmission in a study of Cryptococci where the authors suggest that EV-mediated communication might represent a novel mechanism of virulence that might be common in other infectious species as well [26]. Our experiments show that cells treated with PPF alone do not readily form EVs (Figs. 2cii, iii and iv) and it is possible that PPF exposure by itself does not affect normal cellular functions [27]. However, an unusual behavior was observed when PPF-exposed cells were infected with VSV. We observed formation of large EVs containing mitochondria as well as high levels of VSV. We postulate that PPF exposure alters normal cellular membrane behavior in the presence of VSV. The concept that PPF can induce our immune cells to over-express EVs that support enhanced viral replication is troubling. Could these EVs bypass the immune system and enable vertical transmission of viruses? Is this the reason why Northeast Brazil with the inordinately high number of microcephalic infants and high incidence of larvicide usage in drinking water supplies [28] showed a higher rate of ZIKV-associated microcephaly than other parts of Brazil or anywhere else in the world? We cannot conclusively say. However, our results warrant a closer look at PPF and possibly other pesticides and their impact on our cells and on viral replication, especially that of mosquito borne viruses such as ZIKV. We hope to explore PPF treatment of VSV-GFP infected cells using HeLa cells and trophoblasts.

## Conclusions

Keeping in mind the potential undesirable and even harmful side effects of PPF, it is important to explore alternative options for mosquito eradication until the underlying mechanisms of pesticide alteration of the lipid microenvironment of mammalian cells is elucidated. These options may include the genetically modified (GM) mosquito species, such as the Oxitec mosquito [29], the soil bacterium Bti (*Bacillus thuringiensis* serotype israelensis), which is a mosquito larvicide [30] and *Wolbachia*-infected mosquitoes, which do not support replication of ZIKV, WNEV, Chikungunya virus and DENV, potentially reducing outbreaks due to these viruses [31–33].

It may not be possible to completely prevent the next mosquito-borne viral outbreak. However, proactive ongoing and ecological mosquito control programs will prove effective for the minimization of detrimental outcomes. We need to rigorously and scientifically address whether pesticides can potentially alter our cells and facilitate the vertical transmission of a teratogenic pathogen, such as ZIKV. A recent study demonstrated the use of a network-based model using an integrative systems biology approach as a screening step in the assessment of potential neurotoxicity of chemicals, utilizing PPF as an example [34]. The authors of that study claim low cost and speed of analysis as factors in favor of their approach. Regardless of the methodology used to screen pesticides prior to their use, one must now be wary of their impact on human cells, specifically, human cell membrane lipid composition, which in turn, could impact viral replication as shown in the present study.

## Data Availability

All data generated or analyzed during this study are included in this published article.

## Abbreviations

ZIKV: Zika virus
VSV: Vesicular stomatitis virus
GFP: Green fluorescent protein
PPF: [(2-[1-methyl-2-(4-phenoxyphenoxy) ethoxy] pyridine) or pyriproxyfen]
EV: Extracellular vesicle
